# The Psychological Well‐being of Older Adults With and Without Cognitive Impairment During the COVID‐19 Pandemic

**DOI:** 10.1002/alz70857_106917

**Published:** 2025-12-25

**Authors:** Cassandra Sorin, Natalie A. Phillips

**Affiliations:** ^1^ Concordia University, Montreal, QC, Canada

## Abstract

**Background:**

During the COVID‐19 pandemic, older adults were considered vulnerable to declines in well‐being, especially those with preexisting cognitive impairments. However, qualitative studies have focused on the resilience of cognitively‐unimpaired older adults during the pandemic. Therefore, the present study employed both quantitative and qualitative measures of well‐being to elucidate the pandemic experiences of Canadian community‐dwelling older adults with a range of cognitive profiles, including those with mild cognitive impairment (MCI) and dementia.

**Method:**

Participants were drawn from the Comprehensive Assessment of Neurodegeneration and Dementia (COMPASS‐ND) Study, including the following cognitive profiles: Cognitively unimpaired (CU; *n* = 59, n_female_=45, M_age_=72), Subjective Cognitive Decline (SCD; *n* = 26, n_female_=20, M_age_=72), Parkinson's Disease (PD; *n* = 45, n_female_=19, M_age_=70), Mild Cognitive Impairment (MCI; *n* = 143, n_female_=44, M_age_=75), and Alzheimer's Disease (AD; *n* = 62, n_female_=21, M_age_=75). During the pandemic, participants completed a questionnaire on COVID‐19 experiences, which included the Geriatric Depression Scale (GDS, max. score=30) and the seven‐item Generalized Anxiety Disorder Scale (GAD‐7; max. score=21). Also, a COVID‐19‐specific version of the Impact of Events Scale ‐ Revised (IES‐R; max score=84) assessed behaviours associated with stress and trauma.

**Result:**

In the preliminary quantitative analysis, GDS scores (M=7.3, SD=5.9) were higher for females than males (M_female‐male_=1.6, F=3.911, *p* = 0.049, η^2^ = 0.011), and the diagnostic groups also differed (F=6.038, *p* <0.001, η^2^ = 0.068): CU adults (M=4.5, SD=4.5) had lower scores than participants with MCI (M=7.5, SD=5.8), AD (M=8.3, SD=5.9), and PD (M=8.8, SD=7.2), but not SCD (M=7.2, SD=5.7). On the GAD‐7 (M=2.6, SD=3.6) the scores of female participants were higher than those of male participants (M_female‐male_=1.1, F=4.123, *p* = 0.043, η^2^ = 0.012), but diagnostic groups did not significantly differ (M_diff_ ≤1.8, F=1.424, *p* = 0.226, η^2^ = 0.017). On the COVID‐19‐specific IES‐R (M=10.8, SD=9.9), the main effects of diagnostic group (M_diff_≤3.4, F=1.374, *p* = 0.243, η^2^ = 0.017) and sex (M_female‐male_=1.7, F=1.440, *p* = 0.231, η^2^ = 0.004) did not yield statistically significant results.

**Conclusion:**

Contrary to prior reports, mean scores on measures of psychological well‐being suggested that this sample of older adults experienced relatively low levels of pandemic‐related distress, regardless of differences in cognitive functioning. Future analyses will further clarify these findings in light of participants' qualitative data, physical activity, and level of social support during the pandemic.

